# Effect of oxandrolone therapy on adult height in Turner syndrome patients treated with growth hormone: a meta-analysis

**DOI:** 10.1186/s13633-015-0013-3

**Published:** 2015-08-26

**Authors:** Nicole M. Sheanon, Philippe F. Backeljauw

**Affiliations:** Division of Endocrinology, Cincinnati Children’s Hospital Medical Center, 3333 Burnet Ave MLC 7012, Cincinnati, OH 45229 USA; Department of Pediatrics, University of Cincinnati College of Medicine, Cincinnati, OH USA

**Keywords:** Turner syndrome, Oxandrolone, Growth hormone, Height

## Abstract

Turner syndrome is a chromosomal abnormality in which there is complete or partial absence of the X chromosome. Turner syndrome effects 1 in every 2000 live births. Short stature is a cardinal feature of Turner Syndrome and the standard treatment is recombinant human growth hormone. When growth hormone is started at an early age a normal adult height can be achieved. With delayed diagnosis young women with Turner Syndrome may not reach a normal height. Adjuvant therapy with oxandrolone is used but there is no consensus on the optimal timing of treatment, the duration of treatment and the long term adverse effects of treatment. The objective of this review and meta-analysis is to examine the effect of oxandrolone on adult height in growth hormone treated Turner syndrome patients. Eligible trials were identified by a literature search using the terms: *Turner syndrome, oxandrolone*. The search was limited to English language randomized-controlled trials after 1980. Twenty-six articles were reviewed and four were included in the meta-analysis. A random effects model was used to calculate an effect size and confidence interval. The pooled effect size of 2.0759 (95 % CI 0.0988 to 4.0529) indicates that oxandrolone has a positive effect on adult height in Turner syndrome when combined with growth hormone therapy. In conclusion, the addition of oxandrolone to growth hormone therapy for treatment of short stature in Turner syndrome improves adult height. Further studies are warranted to investigate if there is a subset of Turner syndrome patients that would benefit most from growth hormone plus oxandrolone therapy, and to determine the optimal timing and duration of such therapy.

## Introduction

Turner syndrome (TS) is defined as the complete or partial absence of the second X chromosome in a phenotypic female. The most common features of TS are short stature and gonadal failure. Turner syndrome occurs in 1/2000 live female births. Short stature is one of the cardinal findings in TS, due to haploinsufficiency of the SHOX gene (short stature homeobox-containing gene on the X-chromosome). Growth failure can be seen in early childhood and is usually obvious by 4 years of age. Mean adult height of TS patients without growth hormone (GH) therapy is about 21 cm shorter compared to healthy female adults [1, 2]. Since 1997, the standard treatment for short stature in TS patients has been recombinant human GH. When GH therapy is commenced in early childhood, TS girls can achieve adult height within the normal range [3, 4]. With delayed TS diagnosis, adult height achieved will likely be compromised due to the shorter time period available for catch-up growth. In addition, TS patients often have delayed, incomplete, or absent puberty, and the lack of a pubertal growth spurt further contributes to their adult short stature [5].

Oxandrolone, in addition to GH therapy, has been used as a treatment in TS girls diagnosed at a later age. Oxandrolone, a synthetic anabolic steroid and derivative of testosterone, improves growth by acting directly at the growth plate, and by increasing IGF-I concentrations [6–8]. Oxandrolone has been shown to increase height velocity in girls with TS when combined with GH therapy [3]. The long term beneficial effect of oxandrolone on adult height in TS women has been debated due to the small sample size of previous studies, varied results in adult height outcomes, and the concern of virilization due to this treatment [8–13]. A recent review paper summarized results of three placebo-controlled, double-blind, randomized trials investigating the safety and efficacy of oxandrolone in GH-treated girls with TS [14]. The authors concluded that oxandrolone can be used in TS girls who are severely short or have a poor response to GH despite good compliance. They recommend a dose of 0.03–0.05 mg/kg/day starting at the age 8–10 years.

The purpose of our meta-analysis was to conduct a comprehensive statistical review of all available data on adult height in TS girls treated with GH plus oxandrolone versus GH therapy alone. The advantages of such a meta-analysis are a larger sample size, larger statistical power, and an improved estimate of the true effect size.

## Methods

A comprehensive literature search was conducted to identify all Randomized Control Trials (RCTs) that investigated adult height in GH-treated TS girls also treated with oxandrolone versus placebo. Relevant trials after 1980 were included. Studies before 1980 were excluded because GH was not introduced until 1985. The following databases were searched using the key words “Oxandrolone” AND “Turner Syndrome”: The Cochrane Database, MEDLINE, EMBASE, and PUBMED. The search was limited by language (English), type of subjects (human), date (1980 to present), and type of trial (RCT). The decision to include only RCTs was made in order to improve the validity of the results and to ensure that only high-quality studies were used. The database search was repeated several times using the combinations of keywords and MeSH terms are listed in Table 1. Bibliographies of the 26 initial articles were reviewed for other relevant articles.

**Table 1:** Keywords and MeSH terms used for literature search

Adolescent
Anabolic Agents/therapeutic use
Androgens/adverse effects
Androgens/therapeutic use
Body Height/Physiology
Child
Double-Blind Method
Female
Final Height
Growth Disorder/drug therapy
Human Growth hormone/adverse effects
Human Growth hormone/therapeutic use
Oxandrolone/therapeutic use
Puberty
Time Factors
Turner Syndrome/drug therapy
Turner’s Syndrome/Ullrich-Turner syndrome

Participants were children and adolescents with TS. The diagnosis of TS was confirmed by karyotype. All of the studies excluded participants with Y chromosome material in the karyotype. Participants were also excluded if they had additional endocrine or metabolic disorders that could affect statural growth except for treated primary hypothyroidism.

The main intervention was oxandrolone. All participants were also on GH either at the same time and/or for several years prior to oxandrolone. All TS subjects were treated with estrogen to induce puberty if clinically indicated (some participants had spontaneous puberty). We felt that it was acceptable to include these patients as long as estrogen replacement was provided in a physiologic manner. The comparison of interest was: GH plus oxandrolone versus GH plus placebo.

The main outcome was growth and included studies which reported adult height, near-adult height, and gain in height. Adult height is the gold standard measure of the efficacy of GH treatment. At the end of the evaluation of all articles only four studies had adult height data and only three studies reported gain in height.

The quality of the articles was assessed formally using the Jaded scale. All of the included studies received a score of 3 or higher. Studies were excluded if: 1- there was not a GH alone or GH + placebo group, 2- adult height or near-adult height data were not reported, or 3- the study had a Jaded score of less than three.

Data was extracted by the primary author. The extraction was completed in duplicate to ensure the data was extracted correctly. There were no discrepancies identified between the two different data extractions. The data extracted included: number of total patients, number of patients in each group, dose of therapies (GH, oxandrolone, placebo, and estrogen), length of therapies, initial height, final height, predicted adult height, and adult height gain. Additional data extracted, when available, included karyotype, baseline skeletal age, and mid-parental height.

The principal measure of effect was mean difference as the data was continuous. The primary outcome was adult height. Studies that did not have adult height data were excluded. The secondary outcome was adult height gain. Studies were excluded from the secondary analysis if adult height gain was not reported or if adult height gain could not be calculated from the data provided.

Two different statistical models were used: Fixed Effects model and Random Effects Model. Heterogeneity between studies was assessed using Cochran Q statistic with a p value of less than or equal to 0.05 indicating significant heterogeneity. Secondary analysis was not indicated as the degree of heterogeneity was not significant. A sensitivity analysis was performed by running the Random Effects model using different combinations of the included studies to identify if one study had a greater effect on the results.

## Results

**Fig. 1:**
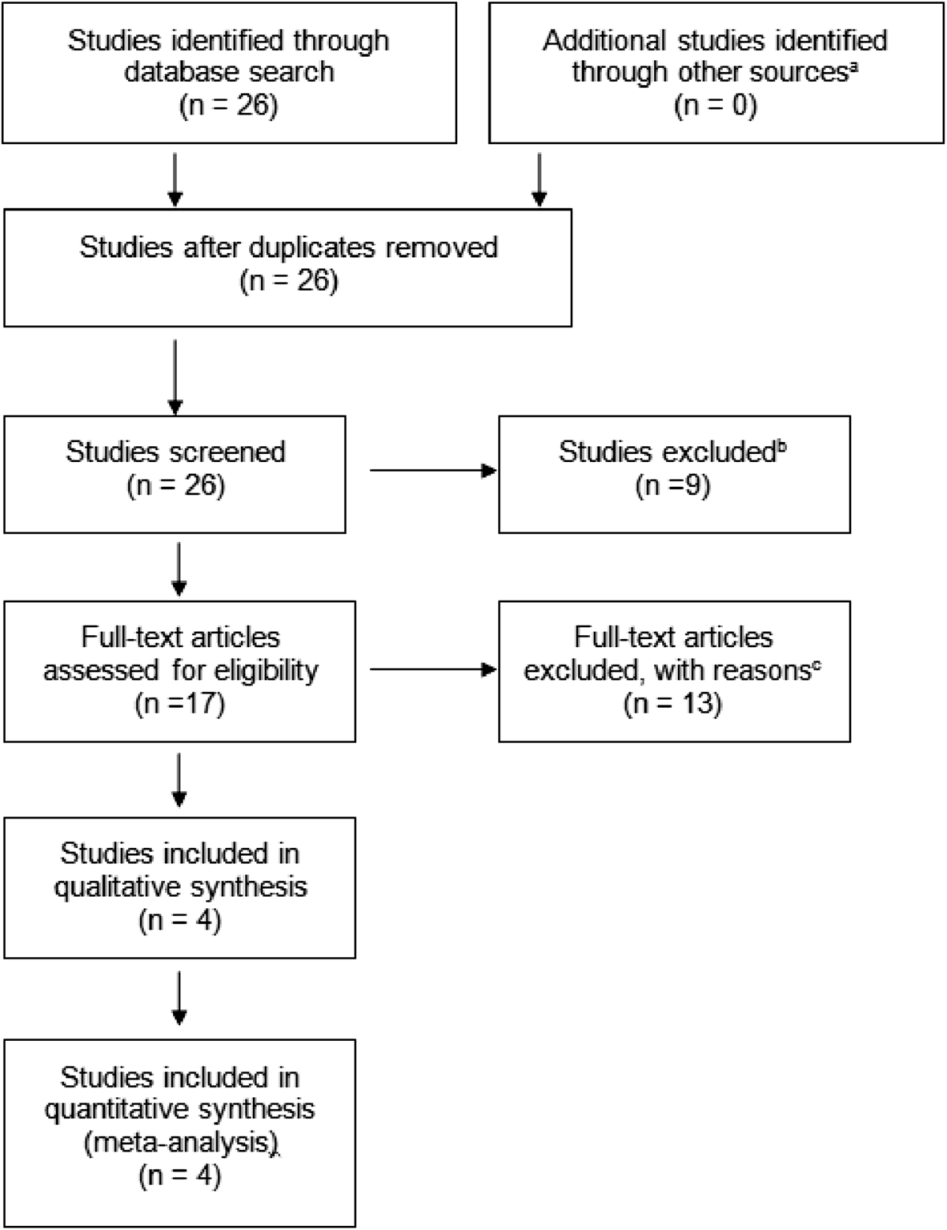
Flow diagram of article selection. The initial literature search identified 26 articles. Nine articles were excluded because they were not evaluating adult height. An additional 13 articles were excluded because adult height data was not available, not all subjects were treated with growth hormone, or the study included the same cohort as a selected study. ^a^ = other sources included asking authors in the field if they were working on any current studies in this area and footnote chasing (looking at references of the 26 articles to identify more articles). ^b^ = 9 studies were excluded because the primary aim was not evaluating final height/near final height, ex. Effect of oxandrolone on glucose metabolism, on voice frequency, on psychological and behavioral characteristics, on body proportions, gonadotropin pulsatility, lipoprotein a, arithmetic learning disability, cognition, and thyroid hormone parameters (reference # 15–23). ^c^ = 13 studies total were excluded for the following reasons: 3 studies did not have final height or near final height data (ref #12,13,31), in 2 studies not all subjects were treated with GH (ref #7, 11), 7 of the studies involved the same cohort/population as the 4 chosen studies (ref #24-30), and 1 study had no control group on GH only (ref#32)

The literature search resulted in 26 articles (see Fig. 1). Upon initial review of the abstracts 9 studies were excluded because the outcome being investigated was not adult height [15–23]. Full-text articles were obtained for the remaining 17 articles. Of these 13 additional articles were excluded because they either involved studies on the same patient cohort reported at different time points [24–30], or the subjects were not all treated with GH [7, 11], or adult height data was not available [12, 13, 31], or there was no control group for GH alone or GH plus placebo [32]. Four articles met criteria and were included in the meta- analysis [3, 33–35]. Bibliographies of these four selected papers were reviewed for other relevant articles; no additional articles were discovered.

The first study was a double-blind, dose–response investigation in which patients were treated with GH from baseline either combined with placebo or oxandrolone (0.03 mg/kg/d or 0.06 mg/kg/day) started at 8 years of age [33]. At 12–13 years of age all the patients were started on estrogen (except those that progressed spontaneously into puberty). A second study was a prospective RCT in which patients who were on GH were placed on placebo or oxandrolone 0.05 mg/kg/day (max dose 2.5 mg) at an average age of 10.2 years and near adult height was obtained [34]. Patients were randomized to receive estrogen at 12 or 14 years of age (except those with spontaneous puberty). Rosenfeld published several RCT studies on the same cohort of patients. The first phase was observation on no medication, oxandrolone alone, GH alone, or oxandrolone + GH. In the second phase the GH-only group continued on GH while the other 3 groups were placed on GH plus oxandrolone. The initial dose of oxandrolone was 0.125 mg/kg/day but this was decreased after the first year to 0.0625 mg/kg/day due to virilization. Oxandrolone was started at a mean age of 9.2 years. Estrogen therapy was delayed in all subjects until a chronological age of 14 years. Patients were followed until they reached adult height [3]. The fourth study was also a prospective RCT of patients (average age of 10.3 years) on GH alone or GH with oxandrolone (0.1 mg/kg/day). Puberty was induced at a mean age of 14.9 years. The patients were followed for 5 years and only 47/91 had reached adult height at time of publication [35]. Individual study results are summarized in Table 2.

**Table 2:** Individual study results. Descriptive data, Mean and standard deviation for adult height and net height gain

Study	Sample size	Mean age baseline yrs (SD)	Mean BA baseline yrs (SD)	Mean MPH	Intervention	Dose of Ox (mg/kg/day)	Mean time on Ox yrs	Mean AH cm (SD)	Mean Ht gain Cm (SD)	Age at estrogen initiation Yrs (SD)	Puberty Sp (%)
Menke [33]	42	8.5 (4.0)	8.1 (3.6)	NR	GH + Ox	0.03	6	156.7 (7.2)	9.5 (4.7)	12.8 (0.9)	21
	36	9.1 (3.5)	8.8 (3.4)	NR	GH + Ox	0.06	6	156.5 (5.8)	8.3 (4.7)	12.7 (0.9)	24
	42	9.4 (3.8)	9.0 (3.4)	NR	GH + Placebo		-	155.6 (5.4)	7.2 (4)	12.9 (1.0)	25
Gault [34]	47	10.2 (1.1)	NR	NR	GH + Ox	0.05	5	151.06 (4.53)	-	12 (*N* = 12) 14 (*N* = 24)	23
	52	10.3 (0.9)	NR	NR	GH + Placebo		-	149.72 (6.75)	-	12 (*N* = 17) 14 (*N* = 28)	13
Rosenfeld [3]	45	9.9 (2.3)	8	162.5 (4.1)	GH + Ox	0.125^a^	6	152.1 (5.9)	10.3 (4.7)	14.9 (0.9)	NR
	17	9.1 (2.1)	8	164.5 (3.7)	GH + Placebo		-	150.4 (5.5)	8.4 (4.5)	15.2 (0.9)	NR
Stahnke [35]	33	11.8 (2.0)	9.9 (1.0)	166.9 (4.0)	GH + Ox	0.1^b^	4	155.1 (4.5)	7.9 (3.8)	14.9	NR
	38	11.7 (1.8)	9.9 (1.1)	163.6 (4.4)	GH + Ox	0.1^c^	-	152.8 (3.8)	6.4 (3.5)	14.9	NR
	20	11.5 (1.2)	10.3 (0.5)	165.1 (5.5)	GH + Placebo		-	151.7 (3.1)	3.6 (2.6)	14.9	NR

*AH* adult height, *SD* standard deviation, *Ox* oxandrolone, *Ht* height, *BA* bone age, *MPH* mid parental height or target height, *NR* not reported, *Sp* spontaneous

^a^Dose cut in half after the first two years of therapy

^b^Dose decreased to 0.05 mg/kg/day after the first year

^c^transient oxandrolone group, given ox for < 1 year due to side effects

Virilization was the main side effect of oxandrolone that was reported. Girls reported voice deepening, hirsutism, and mild clitoromegaly. In the study by Menke the side effects were reported in 5 % of girls on placebo, 16 % of girls on oxandrolone 0.03 mg/kg/day and in 42 % of girls on oxandrolone 0.06 mg/kg/day [33]. Seven subjects on the higher dose and 1 subject on lower dose oxandrolone discontinued treatment due to the virilization effects. In the study by Gault there were no reports of virilization on a dose of 0.05 mg/kg/day of oxandrolone [34]. Similarly, in the studies by Rosenfeld there were minimal side effects on oxandrolone 0.0625 mg/kg/day, but the dose was reduced from 0.125 mg/kg/day after the first year due to 20 % virilization [3, 27]. In the study by Stahnke there were also reports of virilization (clitoromegaly in 19/44 and deepening of voice in 7/44) but the subjects were on a higher dose of oxandrolone (0.1 mg/kg/day). Considering the data together there is a dose-dependent side effect of virilization with minimal effect with doses of oxandrolone less than 0.06 mg/kg/day.

In each study, for those subjects who did not have spontaneous puberty estrogen was initiated between 12 to 15 years of age. The subjects in the Menke study were started between the age of 12 and 12.99 years on 17-β Estradiol 5 μg/kg/day orally and increased to 10 μg/kg/day after 2 years. Cyclic progesterone was added after at least 2 years of estrogen therapy [33]. The subjects in the Gault study received oral ethinylestradiol starting either at age 12 or 14 years in the following manner: year 1, 2 μg; year 2, 4 μg; year 3, four months of each of 6, 8, and 10 μg. Oral progesterone was started at 15 years of age in all subjects [34]. In the Rosenfeld study estrogen therapy was delayed until a minimum chronological age of 14 years. Oral conjugated estrogens were started at 0.3 mg/day and increased to 0.625 mg/day after 6 months. At 1 year progesterone was added [3]. Finally, in the Stahnke study puberty was induced at a mean age of 14.9 years with estradiol valerate 0.2 mg for the first 6 months and 0.5 mg for the second 6 months. During the second year estradiol was increased to 1 mg and progesterone was started [35]. Each study initiated and titrated estrogen using a slightly different method, but they all used oral estrogens and increased the dose in an incremental fashion in order to simulate natural puberty.

### Model results

**Fig. 2:**
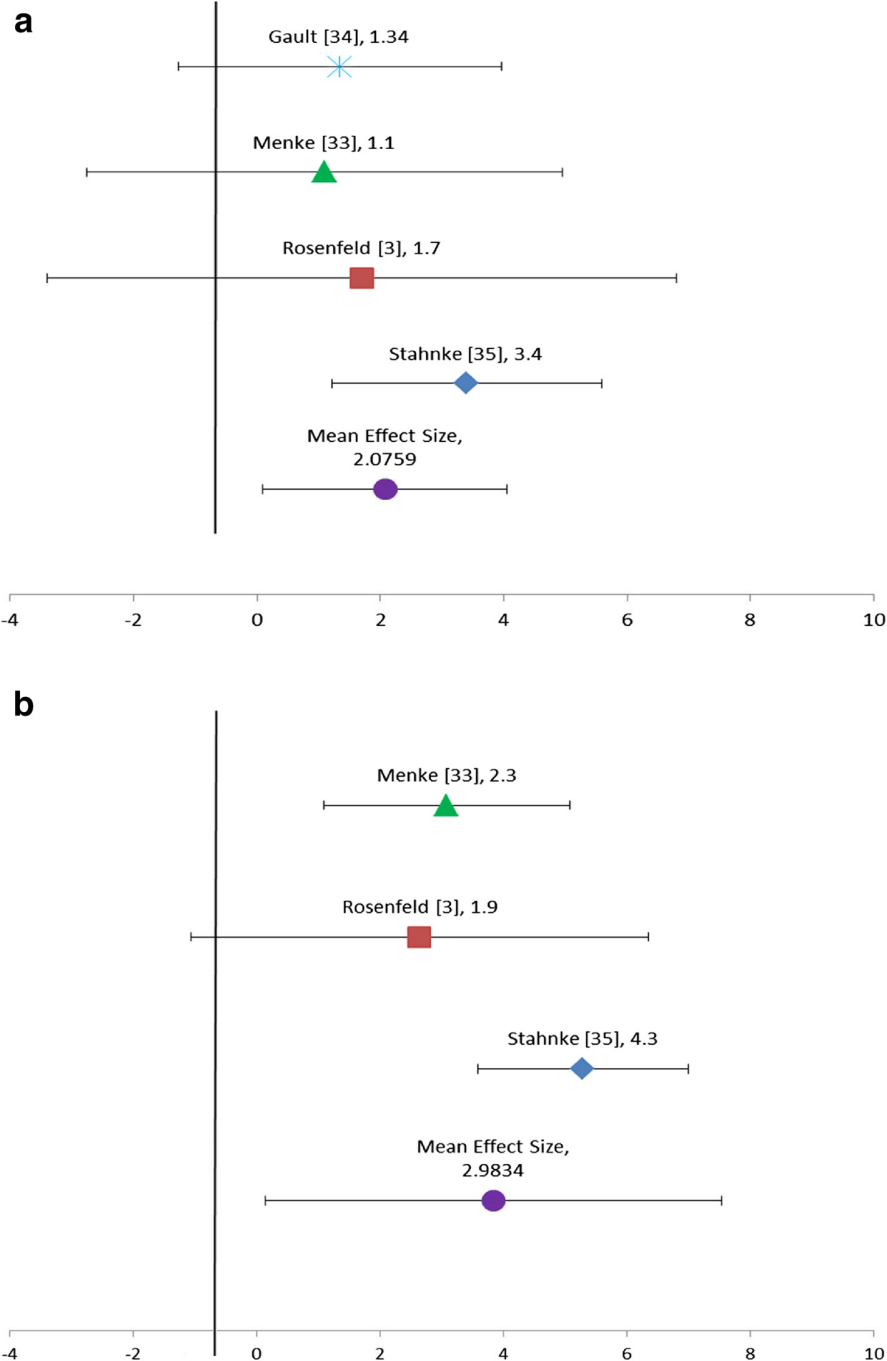
**a** Adult height: effect size and 95 % confidence interval. Forest Plot of effect size for each article and mean effect size for adult height. **b** Height gain: effect size and 95 % confidence interval. Forest Plot of effect size for each article and mean effect size for height gain

The Fixed Effects model (FEM) for adult height yielded a population effect size of 2.0621 cm with a standard error of 0.6441 and a 95 % confidence interval of 0.01239–4.1118. The between-study variance was 0.1055. The Random Effects Model (REM) yielded a population effect size of 2.0759 with a standard error of 0.6212 and a 95 % confidence interval of 0.09882–4.0529. The between-study variance was 1.0900. These results are shown in the Forest Plots in Fig. 2a and b. The effect size for each individual study for adult height and net height gain are listed in Table 3 along with the variance and confidence intervals.

**Table 3:** Adult height and height gain effect size, variance, and confidence intervals

**Adult height**				
Study	Effect Size	Variance	Upper CI	Lower CI
Menke [33]	1.1	1.93	4.957	−2.757
Gault [34]	1.34	1.31	3.966	−1.286
Rosenfeld [3]	1.7	2.55	6.806	−3.406
Stahnke [35]	3.4	1.09	5.588	1.212
Summary	2.0759	0.6212	4.0529	0.09882
**Height Gain**				
Study	Effect Size	Variance	Upper CI	Lower CI
Menke [33]	2.3	0.91	4.114	0.486
Rosenfeld [3]	1.9	1.68	5.264	−1.464
Stahnke [35]	4.3	0.78	5.851	2.749
Summary	2.9834	0.7796	6.3378	−0.371

Heterogeneity was assessed using Cochrane’s Q. The value was 2.5707 with a p value of 0.46 (not significant). A secondary analysis was not performed because there was no significant heterogeneity. The lack of heterogeneity implies that the REM can be used because the difference between the FEM and REM is trivial.

Sensitivity analysis was performed by running the REM and eliminating one study each time to assess if one study was contributing more than the others. The effect size for all 4 studies was 2.0759. The effect size after eliminating the Gault et al. study was 2.3325, after eliminating the Menke et al. study was 2.2966, after eliminating the Rosenfeld et al. study was 2.0950, and after eliminating the Stahnke et al. study was 1.3501.The effect sizes during the sensitivity analysis indicated that the study by Stahnke et al. [35] may have contributed more to the effect size compared to the other studies.

## Discussion

This is the first and only meta-analysis examining the effect of oxandrolone therapy on adult height in GH-treated TS girls. Our findings suggest that oxandrolone may have a positive effect on adult height and height gain when used in combination with GH. The effect size may have been larger if the studies had been more similar in the additional therapies provided (GH and estrogen). However, the lack of heterogeneity found between the studies supports that the differences in dosing and timing of GH and estrogen may not be significant.

Our meta-analysis supports the conclusions from a recent review by Sas et al. [14]. In that paper, data reviewed from three published randomized, placebo-controlled, double-blind studies (which were also included in our meta-analysis) showed that the addition of oxandrolone to GH therapy results in an increase in height velocity and yields a modest increase of adult height [13, 33, 34]. The conclusion was that modest doses of oxandrolone (0.03–0.05 mg/kg/day starting from the age of 8–10 years) were well-tolerated, and that the most important safety issues were enlargement of the clitoris, voice deepening and (temporary) delay of breast development. The authors elected to provide their consensus recommendations after comparing results from three studies only, because of the limited number of well-executed studies, differences in oxandrolone doses used, and because of variations in timing of other therapeutic interventions (GH, estrogens).

The additional strength of our meta-analysis is that the effect sizes for adult height and net height gain were consistent across all studies, indicating that the summary effect size is robust. This can be seen graphically in the Forest plots (Fig. 2a and b). There was moderate discrepancy among the effect sizes in the height gain data, possibly due to the smaller number of studies included. This makes the overall effect size less impactful and the wider confidence interval indicates that this effect size may not be as significant as in the results for the final adult height. The other strength of the meta-analysis is that multiple models were used to analyze the data and the results were similar across all models.

We acknowledge there are some limitations to our meta-analysis approach. There are only four studies included and we may not know the true dispersion of the data. The subjects in each study were on varying doses and duration of GH, which may influence adult height. All of the studies used similar doses of estrogen to induce puberty, but the timing of pubertal induction was different across studies. This is an important point to recognize, because the timing of pubertal induction may affect adult height as well. This limitation was highlighted during the sensitivity analysis: when the Stahnke et al. study was omitted the effect size decreased by 0.7. In this study, puberty was induced later-giving the subjects more time to grow and they reached a taller adult height. It is unclear if the taller height is due to more time for growth, more time on oxandrolone, or more time on GH. Most likely the taller adult height is a result of a combination of these factors. The other factor which cannot be controlled for is the genetic height potential of the patient. Turner syndrome girls with taller parents are more likely to reach a taller adult height compared to girls who have parents of average height. The final limitation was the inability to include additional studies due to missing adult height data. Specifically the study by Zeger, a randomized, double-blind and placebo controlled study, was not included because the raw mean height data and standard deviation data was not available [13]. In this paper they concluded that “the addition of oxandrolone to GH at mean age of 12 (1.7) years augmented height gain after 4 years of treatment.” Those on oxandrolone grew on average 4 cm more than those on placebo [13]. The height gain is greater in the Zeger study than in our meta-analysis; this could be due to the high drop-out rate and only near adult height (defined as bone age ≥ 13.5 years) was assessed. The decision to omit this and other studies was made in order to keep the statistics robust; however, not including these studies could have led to publication bias.

## Conclusion

In conclusion, we provide additional evidence that in short TS girls, in whom severe adult height compromise can be expected, combined treatment of GH plus oxandrolone should be considered. We concur with the recommendation that treatment can be started around 8–10 years of age and that this should be done at oxandrolone doses ranging between 0.03 and 0.05 mg/kg/day. Because of the relative paucity of well-executed studies with large enough patient numbers, we suggest that further research still needs to be done to determine the ideal timing of oxandrolone initiation, the duration of treatment with oxandrolone, as well as to better evaluate the long term adverse event profile of this intervention.

## Abbreviations

TS: Turner syndrome
GH: Growth hormone
RCT: Randomized control trial
FEM: Fixed effects model
REM: Random effects model
